# Viral control of translation: rise of the ribosome

**DOI:** 10.1128/jvi.01282-26

**Published:** 2026-09-14

**Authors:** Peter S. Shen, Derek Walsh

**Affiliations:** 1 Department of Biochemistry, School of Medicine, University of Utah 12348, Salt Lake City, Utah, USA; 2 Department of Microbiology-Immunology, Feinberg School of Medicine, Northwestern University 12244, Chicago, Illinois, USA; Dartmouth College Geisel School of Medicine, Hanover, New Hampshire, USA

**Keywords:** virus, ribosome, translational control, cryo-EM, ribosomal structure

## Abstract

Viruses have evolved a myriad of strategies to manipulate their host’s protein synthesis machinery, studies of which uncovered many founding principles of translational control. Decades of research have established our now-extensive understanding of how these pathogens target the array of translation factors that recruit ribosomes and regulate scanning, start site selection, decoding, and termination. Meanwhile, the ribosome itself was largely regarded as a code-reading machine that lacks regulatory influence. Recent advances in cryo-electron microscopy and quantitative proteomics have upended this viewpoint by revealing the remarkable conformational and compositional plasticity that gives rise to functionally diverse and translationally selective ribosome populations in mammalian cells. Here, we discuss how viruses are once again at the forefront of this emerging field, often bypassing or rewiring host translation factors while directly controlling the ribosome’s composition and intrinsic motions to selectively promote their non-canonical modes of translation. We offer cautionary perspectives on extra-ribosomal functions of ribosomal proteins, while highlighting the emergence of the 40S head domain as a dynamic platform targeted to control various translation processes, ranging from initiation to frameshifting, that expand viral coding capacity. Overall, we chart the rise of the ribosome from a passive player to an active regulatory hub at the center of viral translational control strategies.

## INTRODUCTION

**Fig 1:**
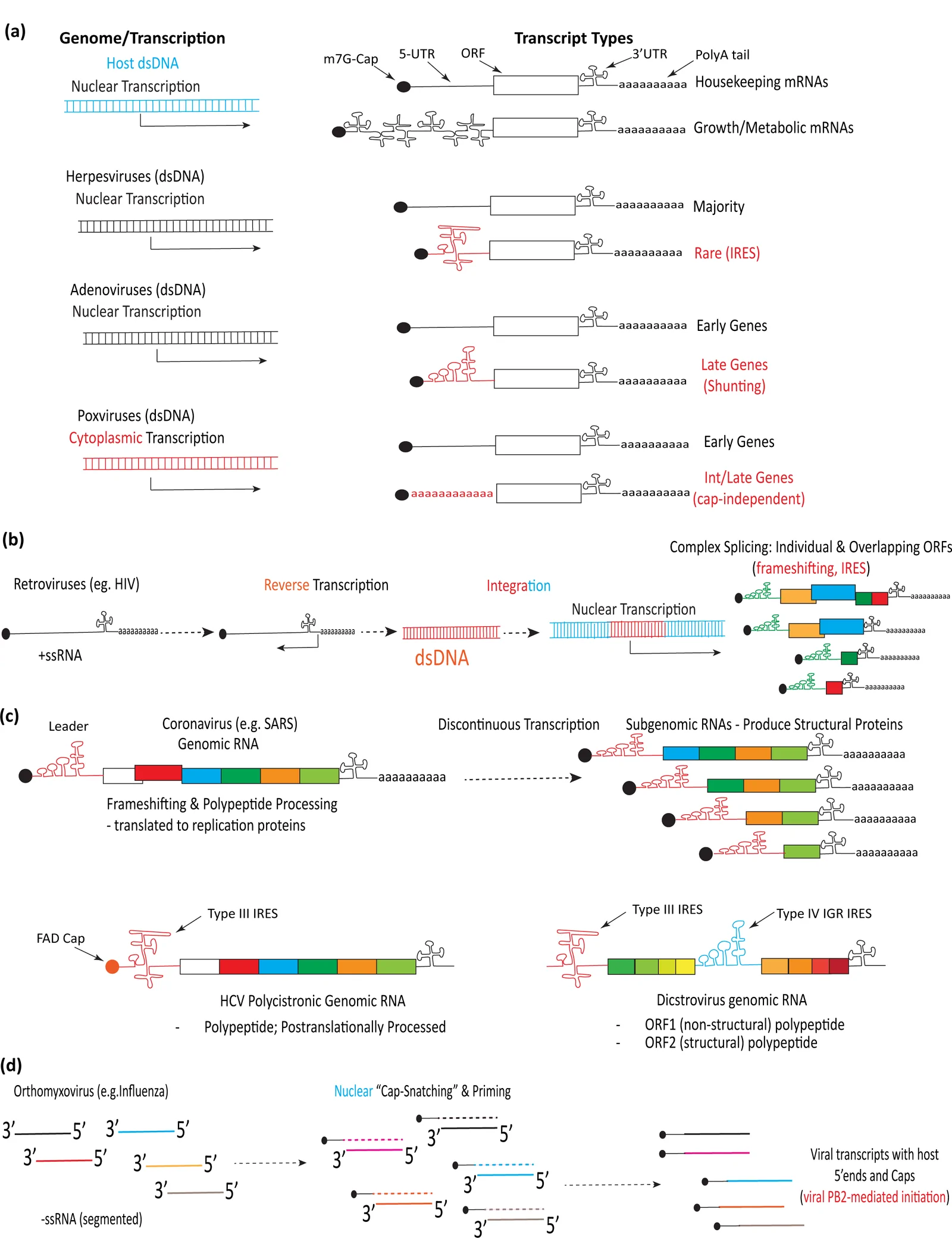
The diversity of viral genome and transcript organizations. Examples of different genome and transcript types across DNA and RNA viruses; note that structural complexity in UTRs is simply illustrative and is not accurately depicted. (**a**) Like their hosts, viruses such as herpesviruses and adenoviruses have dsDNA genomes that are replicated and transcribed in the nucleus. Meanwhile, poxviruses encode their own machinery that enables them to replicate and transcribe their DNA in the cytoplasm. Host cells produce different transcript types, notably using short versus long, structured 5′ UTRs to regulate translation efficiencies. Key structural features of mRNAs are indicated on the representative short host transcript. In this regard, despite genomic similarities, transcripts produced by DNA viruses can differ significantly from their hosts to gain a translational advantage, including the use of internal ribosome entry sites (IRESs), shunting elements, or 5′polyA leaders in their 5′ UTRs (red). (**b**) Retroviruses have capped, positive-sense ssRNA genomes that are reverse transcribed into DNA that integrates into the host genome for transcription. Full-length RNAs serve as both genomes and as polycistronic templates for production of different proteins, including through frameshifting events, while complex splicing produces additional subgenomic mRNA species encoding other viral factors. (**c**) Examples of other positive-sense RNA viruses, which often have polycistronic ORFs that use combinations of frameshifting, reinitiation, and post-translational processing of polypeptides to produce different viral proteins. This includes coronaviruses that use discontinuous transcription to further produce subgenomic RNAs that encode structural proteins, underscoring the extent to which transcription and translation combine to expand their coding capacity. Notably, the HCV genome uses an alternative flavin adenine dinucleotide (FAD)-cap and a Type III IRES for non-canonical initiation, while CrPV lacks a terminal cap and uses two distinct Type III and Type IV IRESs to initiate translation of independent polycistronic ORFs. (**d**) The segmented virus, influenza, steals capped host mRNA 5′ ends to prime transcription of its negative-sense RNAs, but initiates translation using a cap-binding polymerase subunit, PB2, that substitutes for eIF4E.

Viral genomes span a wide range of sizes and organizations, which, in turn, shapes their diverse transcript types and translation strategies (Fig. 1). The largest belong to the nucleocytoplasmic large DNA viruses (NCLDVs), an eclectic group that includes giant pandoraviruses that infect amoebae and *poxviridae*, several of which infect humans. NCLDV genomes can reach several megabases and encode hundreds or sometimes thousands of proteins. Indeed, many are so self-sufficient that they replicate in the cytoplasm using their own DNA replication and transcription machinery. As we descend the size scale, other large DNA viruses, including the *herpesviridae*, whose genomes are hundreds of kilobases, and mid-sized viruses, such as *adenoviridae*, require entry into the nucleus to access host factors that support genome replication and transcription. These double-stranded (ds) DNA viruses largely encode individual open reading frames (ORFs) for each protein, akin to the living organisms that they parasitize, but like many viruses, key structural features of their transcripts diverge to control translation (Fig. 1a). As we enter into the world of RNA viruses, retroviruses, including human immunodeficiency virus (HIV), have a positive-sense single-stranded (ss)RNA genome that is reverse transcribed into dsDNA. This is then integrated into the host genome, transcribed, and spliced into a complex array of mRNA species (Fig. 1b). In most cases, positive-sense ssRNA viruses do not convert to DNA forms. Instead, they often serve as templates for both complex transcription and translation events, and many are polycistronic, meaning multiple ORFs reside in one transcript (Fig. 1c). In some cases, the resulting polyproteins are post-translationally processed, while in others, ORFs can overlap in different reading frames to further maximize coding capacity. Other RNA viral genomes are segmented, meaning they do not consist of a single nucleic acid molecule but instead comprise multiple transcripts encoding different genes (Fig. 1d). Segmentation allows viruses such as influenza to undergo rapid gene swapping or “reassortment” that underlies the continual emergence of new seasonal strains. Moreover, influenza “snatches” the first 10–20 nts of host transcripts to prime transcription of its negative-sense RNAs, which simultaneously adds host 5′ terminal caps to their transcripts (1, 2). Finally, with few exceptions including influenza, most RNA viruses do not require access to the nucleus and replicate entirely in the cytoplasm.

Despite their genomic differences, all viruses have a cytoplasmic phase in their replication cycle. One reason is that no known virus encodes ribosomes, which are the enormous machine-like complexes that host cells use to translate messenger RNAs (mRNAs) into proteins. Moreover, the directional processivity of the ribosome also dictates that all viruses must at some stage produce a positive-sense mRNA that follows the cellular transfer RNA (tRNA)-based triplet coding system. However, the complexity of eukaryotic translational control also offers opportunities for viruses to bend these rules to selectively control both viral and host protein synthesis.

**Fig 2:**
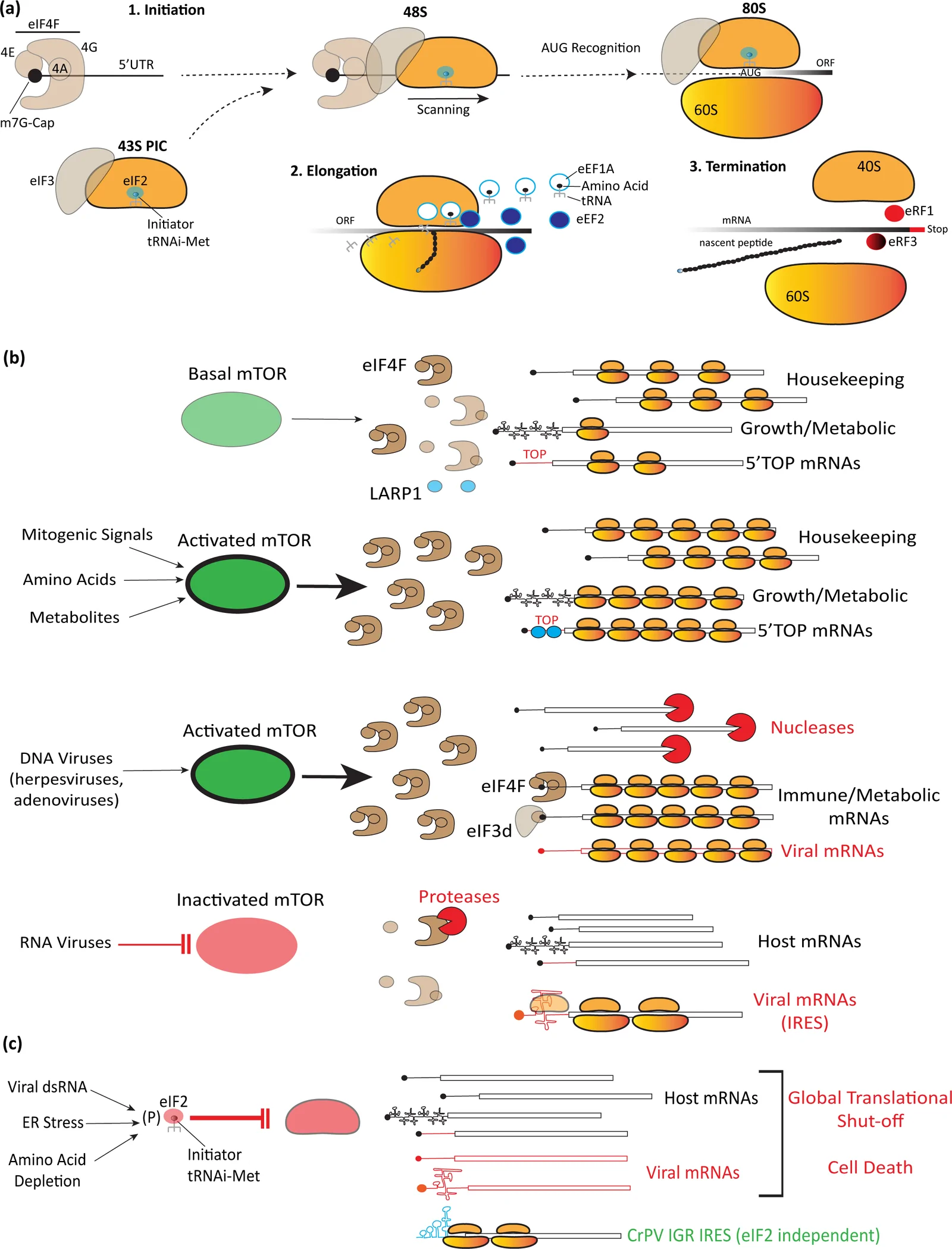
Protein synthesis and translation factor regulation by host cells and viruses. (**a**) The basics of initiation, elongation, and termination*.* Initiation: The 7-methylguanosine cap (m^7^G-cap) on the 5′ end of eukaryotic mRNAs is bound by the eIF4F complex, which consists of the cap-binding protein, eIF4E, the scaffold, eIF4G, and the helicase, eIF4A. The 43S PIC consists of the 40S ribosome bound by numerous eIFs, notably including the eIF2 complex that carries the initiator tRNAi^Met^ and eIF3 that binds eIF4G to form the 48S complex. The bound PIC then scans the 5′ UTR to find the AUG start site, at which point most eIFs depart as the 60S subunit joins to form an 80S ribosome. Elongation: During elongation or decoding, aa-tRNAs are delivered by eEF1A, while eEF2 promotes translocation. Termination: Upon stop codon recognition, eRFs assist in releasing the nascent peptide and separating the mRNA and ribosomal subunits for a new round of translation. (**b**) The role of mTOR in translational control. In resting cells, mTOR activity is low, and mRNAs with structurally complex 5′ UTRs or 5′ TOP elements are translated inefficiently; transparent eIF4Fs represent unassembled complexes lacking eIF4E. In response to environmental stimuli such as mitogens or increased metabolites, activation of mTOR increases eIF4F, thereby greatly increasing the translation efficiency of highly structured mRNA types that rely heavily on eIF4F helicase activity. mTOR also increases the activity of LARP1 to selectively enhance 5′ TOP-mRNA translation. Several DNA viruses activate mTOR, and instead suppress host translation using strategies such as virally encoded nucleases. Meanwhile, increases in both eIF4F and alternative eIF3d-driven m^7^G-cap recognition pathways can increase translation of mRNAs encoding negative immune regulators and metabolic proteins, as well as some viral transcripts. By contrast, RNA viruses can inhibit mTOR or use proteases to degrade eIFs to suppress host translation, while IRES elements sustain viral protein synthesis. (**c**) Several stresses, including viral dsRNA, a transcriptional byproduct of many DNA and RNA viruses, cause phosphorylation of eIF2α, sequestering this critical AUG-recognition factor. This shuts down most forms of host and viral translation, killing infected cells to limit virus spread within the host organism. However, many viruses counteract this critical response; otherwise, they could not replicate. Viruses such as CrPV bypass this response by using eIF2-independent initiation mechanisms. For more details and examples, see the main text and cited reviews.

The canonical mode of mRNA translation proceeds through three primary stages: initiation, elongation, and termination (3) (Fig. 2a). Initiation involves approximately 30 eukaryotic initiation factors (eIFs), which assemble into different complexes with specialized roles. These include eIF4F, which recognizes the 7-methylguanosine (m^7^G)-capped 5′ end of mRNAs and unwinds secondary structures during ribosome recruitment and scanning. Separately, the small 40S ribosomal subunit is assembled into a 43S pre-initiation complex (PIC) containing factors such as eIF2, which delivers the methionine initiator transfer RNA (tRNAi^Met^), and eIF3, a 13-subunit complex that binds mRNA and interacts with eIF4F to load the PIC onto transcripts. The resulting 48S complex then scans the 5 untranslated region (5′ UTR) to identify the start codon, typically the methionine-encoding AUG (4). Once the start site is identified, the large 60S ribosomal subunit joins to form a translationally competent 80S ribosome. Most eIFs dissociate as the 80S enters elongation, wherein it decodes the ORF with the assistance of eukaryotic elongation factors (eEFs); eEF1A delivers aminoacyl-tRNAs, while eEF2 drives tRNA translocation and movement from one codon to the next. During termination, eukaryotic release factors (eRFs) help recognize the stop codon and separate the ribosome, mRNA, and nascent protein.

Translation is the most metabolically demanding process in the cell while also allowing rapid reshaping of the proteome in response to environmental cues. Translational activity is therefore tightly regulated by a wide range of mitogenic, metabolic, and stress signaling pathways. Several of these converge on the kinase mechanistic target of rapamycin (mTOR), which also directly senses amino acid and energy availability (5) (Fig. 2b). One primary way in which mTOR influences translational responses is by stimulating the formation of eIF4F complexes. While mTOR activation increases overall protein synthesis, it also leads to a particularly robust increase in the translation of growth-related and metabolic transcripts, which have long, structurally complex 5′ UTRs that rely heavily on eIF4F availability for efficient helicase-mediated scanning (4). mTOR also enhances translation of mRNAs with 5′ terminal oligopyrimidine (TOP) motifs, many of which encode ribosomal proteins, thereby amplifying translational stimulation (6).

Viruses exploit these complex regulatory pathways in diverse ways, frequently targeting mTOR and eIFs, together with other RNA helicases and regulatory factors (1, 2). Large dsDNA viruses often produce capped mRNAs and activate host cap-dependent translation factors. These include mTOR, eIF4F, and alternative eIF3d-driven pathways (7–9), although this sometimes controls production of host antiviral and metabolic proteins, while eIF4F is not always directly involved in viral mRNA translation (7, 8, 10) (Fig. 2b). Indeed, poxviruses switch to cap-independent modes of translation as infection progresses and disrupt mTOR, in large part to block mTOR-mediated antiviral responses (11–13). Many RNA viruses also disrupt mTOR and eIF4F activity, including through proteolytic cleavage of eIF4F subunits, thereby suppressing antiviral responses and reducing competition for ribosomes. Meanwhile, viral translation is sustained by mRNA structures, discussed later, that bypass dependence on inactivated host factors. Finally, one of the most potent cellular stress and antiviral responses centers around phosphorylation and inactivation of eIF2α, which inhibits eIF2B-mediated nucleotide exchange and depletes active eIF2-GTP-Met-tRNAi complexes (Fig. 2c) (1, 2). eIF2-mediated recognition of the start codon is almost universally used by all viral and host mRNAs, and while this potent shutdown of translation often induces cell death, it very effectively contains the spread of infection.

For much of the modern molecular biology era, translational control by host cells or viruses has been framed through the lens of eIFs, while the ribosome was regarded as a passive code-reading machine. However, this viewpoint has been transformed in recent years. While the dynamic states and intrinsic motions of bacterial ribosomes were uncovered in the late 2000s, more recent advances in high-resolution cryo-electron microscopy (cryo-EM), together with quantitative analyses of ribosome populations, have revealed that eukaryotic ribosomes also occupy a rich landscape of conformational and compositional states that are tightly coupled to translational decisions (14–16). These states are not only consequences of factor binding but also represent functional intermediates that can be selectively controlled. These insights now extend beyond purified systems, with *in situ* approaches combining cryo-focused ion beam (cryo-FIB) milling with cryo-electron tomography (cryo-ET), subtomogram averaging, and, more recently, *in situ* single-particle cryo-EM beginning to map the translation cycle directly within intact cells (15–18). As a result, the ribosome is increasingly recognized as an active regulatory hub with intrinsic motions, subunit differences, chemical modifications, and collision-triggered signaling interfaces shaping translational output, and perhaps nowhere is this more evident than during viral infection.

In this review, we briefly contextualize the already well-established contributions of translation factors (1, 2) and focus on emerging insights into the remarkable ways in which the conformational dynamics of the ribosome are exploited by viruses to control virtually every step in the translation process.

## STRUCTURAL AND COMPOSITIONAL DYNAMICS OF THE RIBOSOME

The mature mammalian ribosome consists of approximately 80 ribosomal subunit proteins (RPs) and four ribosomal RNAs (rRNA), intertwined in the form of a 40S and 60S subunit. Considering this complexity and that mammalian cells contain millions of individual ribosomes, it is perhaps unsurprising that these machines have the capacity to structurally and functionally diversify both within and between cell types (19). Central to this seismic shift in understanding has been the application of cryo-EM and proteomics, which have provided unprecedented insights into the conformational, compositional, and functional plasticity of ribosomes.

### Cryo-EM snapshots of ribosome motion

**Fig 3:**
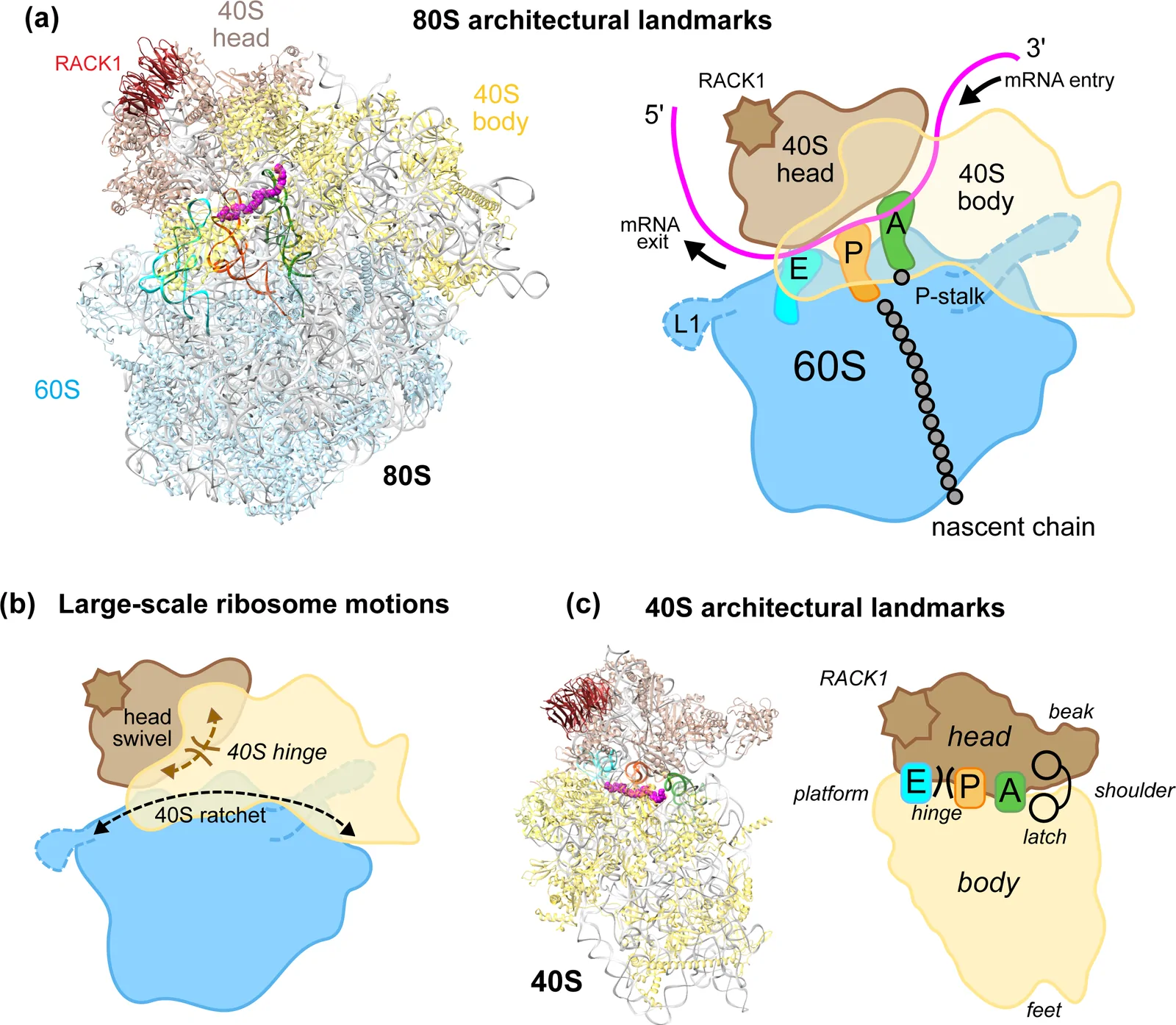
Architectural landmarks and large-scale motions of the eukaryotic ribosome. (**a**) 80S ribosome architecture. Composite cryo-EM model (left) and schematic representation (right) of the eukaryotic 80S ribosome. The small 40S subunit (tan/brown) and large 60S subunit (blue) together form the mRNA channel, decoding center, and peptidyl-transferase center. mRNA (magenta) enters the ribosome at the entry channel on the 40S body and exits near the solvent-exposed surface of the 40S head adjacent to RACK1. The A, P, and E tRNA-binding sites are positioned at the subunit interface, with the P-stalk and L1 stalk contributing to factor recruitment and tRNA dynamics, respectively. The nascent polypeptide emerges through the exit tunnel of the 60S subunit. (**b**) Large-scale ribosome motions. During translation, the ribosome undergoes coordinated conformational changes. Intersubunit rotation involves movement of the 40S subunit relative to the 60S, whereas head swiveling reflects rotation of the 40S head relative to the body about the hinge region. These motions remodel the mRNA channel and tRNA-binding sites and are essential for decoding, translocation, and maintenance of the reading frame. (**c**) 40S architectural features. Cryo-EM structure (left) and schematic (right) highlighting key domains of the 40S subunit. The head, body, platform, shoulder, feet, and beak define the overall morphology of the subunit. The mRNA channel is formed at the interface between the head and body, where rRNA elements (including helices h18 and h34) form a dynamic “latch” that regulates mRNA accommodation. The decoding center lies near the A, P, and E sites, while the hinge region connects the head and body and supports conformational rearrangements during translation. RACK1 is located on the solvent-exposed surface of the head.

A brief structural orientation is useful for framing ribosome dynamics. The 40S subunit contains the decoding center and forms the primary mRNA–tRNA interaction platform, whereas the 60S subunit houses the peptidyl-transferase center (PTC) and the nascent polypeptide exit tunnel (Fig. 3a). Within the 40S subunit, the “body” provides a stable scaffold for mRNA binding and factor engagement, while the “head” undergoes conformational rearrangements.

Building on earlier structural, biochemical, and single-molecule studies, a central contribution of cryo-EM has been the ability to resolve heterogeneous ribosome structures into discrete conformational states that define mechanistic trajectories. During translation, ribosomes cycle through a series of structurally distinct intermediates that are tightly coupled to tRNA movement and factor recruitment. The most fundamental of these motions is inter-subunit rotation, in which the 40S rotates relative to the 60S as tRNAs move through hybrid binding states (14, 20) (Fig. 3b; Movie S1). This large-scale motion coordinates A-site decoding, peptidyl transfer, and eEF2-driven translocation, providing the structural framework for directional mRNA movement.

Superimposed on intersubunit rotation is 40S head swiveling, in which the head rotates relative to the 40S body through their flexible connecting hinge (Fig. 3c). This movement reshapes the mRNA channel and tRNA-binding pockets during the elongation cycle through the tRNA A → P → E sites, thereby coupling local decoding processes to larger-scale control of mRNA threading and reading-frame maintenance. This architectural division also positions the 40S head as a regulatory handle for conformational control, where swiveling can modulate scanning, decoding, and translocation, as well as programmed recoding events such as frameshifting. Moreover, local remodeling of the nascent polypeptide exit tunnel provides an interface for sensing translational status and can communicate the presence of inhibitory sequences to the PTC to adjust or terminate translation accordingly.

Cryo-EM has also provided insights into dynamic extensions of the ribosome. These include flexible rRNA expansion segments (ESs) on both subunits that recruit translational regulators and nucleate inactive ribosomes during stress (21–23). By contrast, the P-stalk is a dynamic multiprotein assembly that extends from the 60S to recruit translational GTPases, which facilitates both initiation as the 80S assembles and subsequent elongation. Structural studies of eukaryotic ribosomes reveal substantial conformational heterogeneity within the P-stalk and its associated rRNA ESs, underscoring their dynamic nature (20, 24, 25), while specific examples of their selective roles in translation are discussed later.

### Ribosome specialization and compositional heterogeneity

Ribosomes can also vary in composition, giving rise to the concepts of functional diversification and “ribosome specialization,” wherein different ribosome types translate different mRNA species (19, 26). While it is now well established that structural heterogeneity can arise in several ways, including differential incorporation of RPs, variation in rRNA modifications, and post-translational modifications (PTMs) such as phosphorylation or ubiquitination, whether and how this results in specialization remains widely debated (27).

Although they do not regulate translational selectivity, two of the best characterized PTMs are ubiquitination and UFMylation of specific RPs that arise from ribosome collisions that alert cells to aberrant translation events, discussed in more detail later (28). Interestingly, the potential influence of phosphorylation of individual RPs on translation is less clear. This is perhaps due to complex context-specific functions and is exemplified by the enigmatic role of RPS6 phosphorylation (29, 30). Beyond RPs, rRNA modifications such as 2′-O-methylation and pseudouridylation cluster at functionally important sites, including the decoding center and inter-subunit bridges, and can be dynamically remodeled during stress or disease (31–33). Structural studies are beginning to resolve these rRNA modifications within cryo-EM reconstructions, strengthening their mechanistic links to altering ribosome structure and function (34–36).

Heterogeneity also arises through the incorporation of distinct paralogs or the absence of specific RPs (19, 37). For example, cell type-specific expression of RPL38 underlies differential translation of HOX mRNAs during cytoskeletal patterning (38, 39). Within individual cells, a limited number of RPs can be expressed at sub-stoichiometric levels, giving rise to functionally distinct ribosome populations with missing subunits (40). In contrast, there are widespread reports of RPs with extra-ribosomal functions, including various viral replication processes. However, these studies need to be interpreted cautiously and in context. Many cell lines have lost the regulatory mechanisms that normal cells use to rapidly degrade extra-ribosomal RPs (41–43). In addition, the 40S and 60S also contain a specific RP that is expressed as a ubiquitin fusion protein (Ub-RPS27a and Ub-RPL40 in humans) that must be processed by deubiquitinases (DUBs) to generate assembly-competent RPs while also supplying the ubiquitin pool (44, 45). If these Ub-RPs are not processed, they are also degraded. Considering their abundance and the propensity of many RPs to bind RNA, degrading extra-ribosomal RPs restricts their functions to the ribosome and likely prevents them from interfering with other biological processes. Indeed, loss of RP homeostasis is associated with cellular transformation and diseases known as ribosomopathies (19, 46). The widespread use of cell lines in research, unless validated as retaining normal RP homeostasis, means that many of these reported extra-ribosomal functions may not operate in the natural context of infection. As such, this review focuses on how viruses directly control the ribosome, while we direct readers to other reviews of RP functions both on and off the ribosome (47, 48).

## VIRAL CONTROL OF TRANSLATION INITIATION

**Fig 4:**
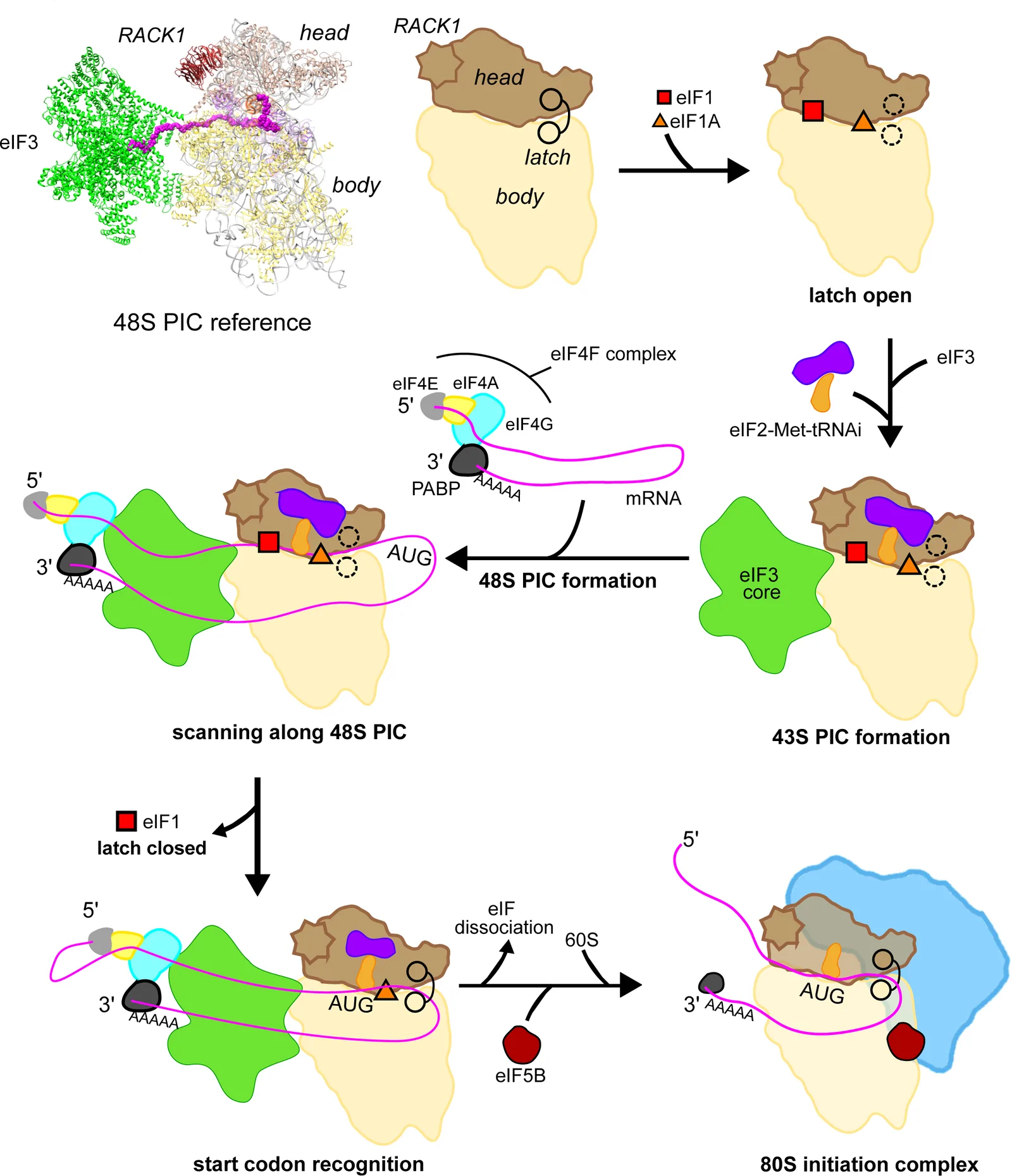
Conformational transitions during cap-dependent translation initiation. Schematic overview of canonical eukaryotic translation initiation, highlighting ribosome conformational states and factor dynamics. A reference cryo-EM structure of the 48S pre-initiation complex (PIC) is shown (top left) to anchor the schematic representations. In the absence of initiation factors, the 40S subunit adopts a latch-closed conformation, in which interactions between rRNA elements (including helices h18 and h34) restrict access to the mRNA entry channel. Binding of eIF1 (red square) and eIF1A (orange triangle) promotes latch opening, generating an mRNA-receptive state. Assembly of the 43S PIC occurs upon recruitment of eIF3 and the eIF2-GTP-Met-tRNA_i_ ternary complex, positioning the initiator tRNA near the P-site. The eIF4F complex (eIF4E, eIF4G, and eIF4A) binds the 5′ m^7^G-capped mRNA, in conjunction with poly(A)-binding protein (PABP), forming a closed-loop mRNP that ensures translation occurs on properly processed mRNAs and stimulates a new round of initiation after termination. Interaction between eIF4G and eIF3 promotes recruitment of mRNA to the 43S PIC, forming the 48S complex. During scanning, the 48S PIC translocates along the 5′ UTR in a latch-open conformation, inspecting the mRNA for an AUG start codon. Upon start codon recognition, the ribosome undergoes a transition to a latch-closed state, accompanied by dissociation of eIF1 and repositioning of initiation factors. This conformational switch stabilizes initiator tRNA in the P-site and promotes eIF5-stimulated GTP hydrolysis by eIF2. Subsequently, eIF2–GDP and other initiation factors dissociate, and eIF5B facilitates joining of the 60S subunit to form the 80S initiation complex, positioning the ribosome for entry into elongation.

Initiation can be viewed as a process of conformational gating wherein eIFs stabilize distinct ribosome states along the pathway from mRNA recruitment to start-codon selection (3) (Fig. 4). During scanning, the 43S PIC adopts an “open” configuration in which the mRNA entry channel is widened and the latch between helices h18 and h34 of the 18S rRNA is loosened, thereby facilitating mRNA accommodation and directional movement. This opening is facilitated by eIF3 and stabilized by eIF1 and eIF1A, which maintain a scanning-competent conformation at the decoding center and head–body interface.

Recognition of the start codon is accompanied by a complex, multifactor-driven transition toward the “closed” state that commits the ribosome to initiation (3, 4). This involves tightening of the latch, eIF5-stimulated GTP hydrolysis by eIF2, and repositioning of the tRNAi^Met^ within the P-site, alongside remodeling at the decoding center. Dissociation of eIF1, conformational changes in eIF1A, and subsequent eIF5B-mediated rearrangements leading to 60S joining accompany this transition, while eIF3 coordinates early PIC assembly with downstream factor rearrangements. Initiation decisions therefore arise directly from ribosome conformational transitions rather than translation factor binding alone. Underscoring this point, several viruses employ alternative initiation factor-independent strategies to directly control these conformational states, ranging from direct modulation by RNA structures to virally encoded kinases that target specific RPs.

### RNA elements that control initiation

Internal ribosome entry sites (IRESs) are the best-characterized RNA elements whereby viruses control initiation through the ribosome. IRESs comprise a broad family consisting of complex stem-loop and hairpin organizations and are broadly classified into four groups based primarily on shared translation factor dependencies. Types I and II retain partial dependence on components of the eIF4F complex, with core distinctions including reliance of Type I IRESs on interactions between the scaffolding subunit of the eIF4F complex, eIF4G, and eIF3 for 40S recruitment and scanning (49). By contrast, the Type II IRES of encephalomyocarditis virus (EMCV), for example, interacts with eIF4G but nevertheless also makes essential, conserved contacts with the ribosome itself. Cryo-EM structures show that the apical domain I cloverleaf contacts RPS18 and RPS15 within the 40S head, while a conserved GNRA tetraloop elsewhere in the IRES structure engages the tRNAi^Met^ (50). These ribosome–RNA interactions stabilize a scanning-arrested, closed conformation of the 40S subunit, involving rearrangements at the latch and head–body interface (51). As such, while traditionally viewed as eIF4G-driven, these IRESs also directly interact with and alter ribosome conformations.

Indeed, a unifying theme emerging from structural studies is that IRESs function as architectural regulators of ribosome dynamics, capable of stabilizing specific conformations. Among these, the most structurally and mechanistically resolved examples are the Type III hepatitis C virus (HCV) IRES and distinct Type III-IV IRESs of dicistroviruses, which together define a spectrum of increasing autonomy from eIFs.

### The Type III IRES of HCV

**Fig 5:**
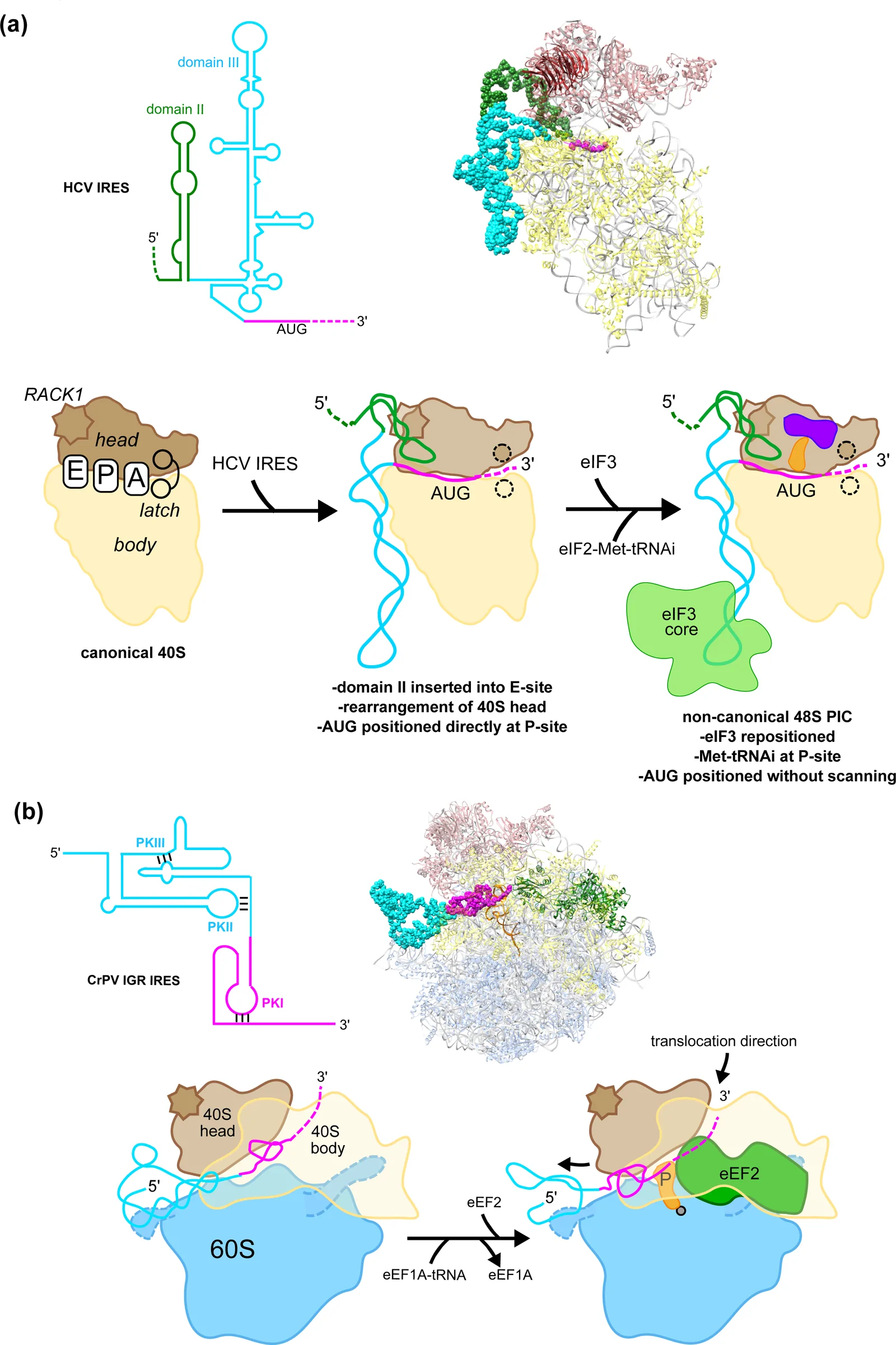
Structural mechanisms of IRES-mediated ribosome recruitment and initiation. Type III and Type IV IRESs illustrate distinct strategies for direct ribosome engagement that bypass canonical cap-dependent initiation pathways. (**a**) Type III IRES (HCV-like). The HCV IRES binds directly to the 40S subunit and the 43S pre-initiation complex, engaging the solvent-exposed face of the ribosome. Domain II extends into the E-site region and induces conformational rearrangements in the 40S head, while Domain III recruits eIF3 in a non-canonical configuration. These interactions bias the ribosome toward conformational states characterized by opening of the mRNA entry-channel latch and partial 40S head swiveling, enabling positioning of the start codon near the P-site without extensive scanning. Initiation proceeds with reduced dependence on canonical initiation factors. (**b**) Type IV IRES (dicistrovirus intergenic region [IGR]). IGR IRES elements from dicistroviruses directly assemble ribosomes in the absence of canonical initiation factors. The IRES binds to 80S ribosomes and adopts a compact fold that mimics tRNA interactions within the decoding center, initially occupying the A site. An eEF2-mediated pseudo-translocation event shifts the IRES into the P-site, freeing the A site for incoming aminoacyl-tRNA and enabling initiation from non-AUG codons. This mechanism exploits intrinsic ribosome dynamics, including inter-subunit rotation and 40S head swiveling, to transition directly into elongation.

The HCV IRES exemplifies how structured RNA engages the 43S PIC to bypass canonical cap-dependent initiation. Early studies demonstrated that the IRES contains spatially distinct binding regions for eIF3 and the 40S subunit (52, 53). Cryo-EM later revealed the IRES docks onto the solvent-exposed face of the 40S subunit, engaging the 18S rRNA expansion segment ES7, with its domain II extending into the E-site region and inducing rearrangements within the decoding center (54–56) (Fig. 5a; Movie S2). In parallel, domains within the apical region of the IRES recruit and reposition eIF3 in a non-canonical configuration that reshapes the PIC (57, 58). Importantly, the HCV IRES biases ribosome conformational equilibria rather than acting solely as a recruitment signal. Cryo-EM revealed multiple conformational sub-populations of HCV IRES–ribosome complexes, highlighting dynamic stabilization of an extended 40S head-swiveled state, opening of the latch region, and persistent engagement of the E-site by domain II (59). These conformations are distinct from elongation-associated swiveling states and are thought to promote accurate positioning of the initiator tRNA while bypassing canonical scanning. Initiator tRNA accommodation is further stabilized by eIF1A, which the HCV IRES actively exploits to remodel the PIC (60). Supporting these structural observations, functional studies show the importance of proteins of the 40S head, including RACK1 on the solvent-exposed surface near ES7, the hinge, and the mRNA exit site, and RPS25 near the mRNA entry channel (48, 61–64). This network of interactions likely underpins the broader control this and related IRESs exert over the swivel motion of the 40S head. Beyond capturing 40S subunits, the IRES can also engage terminating ribosomes (65) and, following initiation, the IRES continues to engage eIF3 to control 80S entry into elongation (66).

### The autonomous Type IV IRESs

Members of the Dicistrovirus family of positive-sense single-stranded RNA viruses, exemplified by Cricket Paralysis Virus (CrPV), encode two distinct IRESs for different polycistronic ORFs (Fig. 1c). The first is a 5′ UTR IRES that requires eIF3 and employs an upstream ORF (uORF) to assemble a functional initiation complex using a multidomain structure that circles and controls the 40S head (67). Remarkably, a second, intergenic region (IGR) IRES initiates translation without any canonical initiation factors (68) (Fig. 5b). To accomplish this, the IGR IRES binds directly to 80S ribosomes and uses an anti-codon stem-loop that mimics tRNA interactions within the decoding center, initially in the A-site. An eEF2-mediated pseudo-translocation to the P-site frees the A-site to then initiate translation from non-AUG codons, typically CGU or CGA (69–74). The IGR IRES also exploits intrinsic ribosome motions, including head swiveling and inter-subunit rotation, to transition into elongation. Interestingly, this IRES can recruit the 40S and 60S subunits simultaneously or sequentially, while eEF2-assisted translocation to elongation influences frame selection (75) and the rate of early elongation steps (76). Studies of related *dicistroviridae,* including Israeli acute paralysis virus and Taura syndrome virus, further highlight the dynamic, “inch-worm” like behavior of IGR IRESs themselves as they control ribosome conformational states (77, 78). As if not extreme enough, the related Halastavi árva virus and Wenling picorna-like virus IRESs adopt an even simpler mode of initiation by directly engaging the P-site and initiating without a prior translocation step (79, 80). The *dicistroviridae*’s mode of initiation bypasses the need for eIF2, a core component of antiviral responses. Moreover, although some IRESs of human viruses can function in yeast (62, 81), the IGR IRES is so factor-independent that it also functions in bacteria (82, 83). Recent sequence mining and functional studies show that many variants of these IRESs exist (84–86), suggesting much remains to be learned about these remarkable RNA elements.

### Other RNA elements and unusual initiation mechanisms

Other viruses use distinct structural elements to control ribosomes and reduce dependencies on eIFs. Among those that have been structurally characterized, a downstream stem-loop (DSL) within the ORF, rather than the 5′ UTR, enables initiation on alphavirus mRNAs even in the presence of phosphorylated eIF2. To do this, the DSL interacts with the 18S rRNA extension, ES6S on the solvent face of the 40S, trapping scanning PICs and placing the upstream initiator AUG directly into the P-site (87, 88).

Highlighting how RNA and DNA viruses differ, IRES elements have only been reported in single transcripts from a few DNA viruses, including small polyomaviruses and some herpesviruses (2). Adenovirus late mRNAs employ an unusual shunting mechanism that bypasses scanning. This is driven by a 200 nt tripartite leader in viral mRNAs that has complementarity with 18S rRNA and which also binds the viral 100K protein that recruits eIF4G (89). While the structural basis of shunting remains unknown, functional studies show that RPS25 is required for shunting and initiation by several IRESs (61), suggesting potential similarities in how these non-canonical modes of initiation operate.

### Viral proteins that modify ribosome selectivity

Although less frequent, some RNA viruses encode proteins to control ribosome dynamics and selective initiation on viral versus host mRNAs. The best characterized is the non-structural protein, Nsp1 of SARS-CoV-2 and related betacoronaviruses, whose C-terminal domain inserts directly into the mRNA entry channel of the 40S subunit (90–102) (Movie S2). Structures of MERS-CoV and bat coronavirus Nsp1 reveal broadly conserved binding strategies across mammals, including humans and bats, while highlighting differences in affinity for distinct initiation-factor contexts (90, 103, 104). Nsp1 binds near the 40S latch and sterically occludes mRNA entry. This also places Nsp1 at the intersection of 40S head swiveling and entry-channel gating, suggesting that Nsp1 also regulates ribosome conformational dynamics. Consistent with this, cryo-EM shows that Nsp1 associates with multiple early initiation intermediates rather than a single static ribosome state. Structures of idle 40S subunits and eIF3-containing PICs indicate that Nsp1 binding is compatible with ribosomes poised for initiation but not yet committed to mRNA accommodation (90, 92). In these assemblies, the 40S subunit adopts conformations associated with an open or partially open mRNA entry channel, and Nsp1 binding appears to stabilize these early initiation-like conformations while preventing the transition to downstream mRNA-engaged states, effectively trapping ribosomes in a translationally inert ensemble. Nsp1-bound ribosomes that engage host mRNAs are also prone to stalling and subsequent mRNA decay (93, 94, 97). Meanwhile, viral mRNAs evade repression through cis-acting features within their 5′ leader sequences, which include a conserved SL1 hairpin that can relieve or bypass Nsp1-induced entry-channel occlusion (105–107). This selectivity also involves N-terminal domains of Nsp1, which bind mRNA and influence Nsp1 interactions with the 40S, whose remodeling favors viral mRNAs (97, 99). Combined, these findings reinforce the idea that Nsp1 does not globally inactivate ribosomes but instead helps reshape the conformational landscape to discriminate between host and viral mRNAs.

Finally, other RNA viruses may employ similar but currently less-well understood strategies. For example, the N protein of Hantavirus also binds both viral mRNA and the 40S head protein, RPS19 (108, 109). While N binding causes conformational changes in RPS19, whether it affects the dynamics of the 40S head remains unknown.

### Customization of ribosomes to control initiation

Larger DNA viruses are more frequently reported to alter ribosome composition through the activity of virally encoded proteins than through RNA elements. At ~235 kb of dsDNA, cytomegalovirus (CMV) has the largest genome of viruses that infect humans. Despite this, CMV does not encode its own translation factors or ribosomes, and instead it translationally upregulates RPs (110, 111). This is driven by the viral protein, uL38, that activates upstream signaling to mTOR, but whether CMV also alters ribosome dynamics or heterogeneity remains unknown. Several NCLDVs that infect amoeba encode various translation factors and complexes, and as many as 70 tRNAs, 20 aminoacyl-tRNA synthetases, segments of 18S rRNA, and several RP homologs (112–116). This raises the possibility that these viral giants may customize ribosomes in unusual ways, potentially through viral rRNAs or RPs replacing their host counterparts.

However, DNA viruses have not been widely reported to alter the RP composition of ribosomes. Instead, evidence points to modulation through PTMs to individual RPs. For example, herpes simplex virus type 1 (HSV-1) infection results in unique phosphorylation events in the 40S subunits RPS2, RPS3a, RPS6, and RPSa (117), as well as the 60S protein RPL30 (117, 118), but whether these modifications regulate HSV-1 translation and what kinases are involved remains unknown. RNAi screens also show that HSV-1 has a lower dependency on RPL3, RPS9, and RPL11 than host translation (119). Combined, these findings hint at potential changes to ribosome structure and selectivity during HSV-1 infection. Another herpesvirus, Kaposi’s sarcoma-associated herpesvirus (KSHV), co-opts the cellular protein BUD23, a conserved S-adenosylmethionine-dependent methyltransferase, where it is recruited to pre-40S complexes and increases 18S rRNA m^7^G1639 modification (120, 121). This is proposed to generate specialized ribosomes that bypass uORFs to increase translation of downstream viral ORFs, although potential effects on ribosome biogenesis and the overall activity of the ribosome pool have not been fully ruled out. KSHV has also been reported to regulate pseudouridylation, which occurs on rRNAs, tRNAs, and several other RNA types (122, 123), but how this influences ribosome biogenesis versus translation remains unknown.

**Fig 6:**
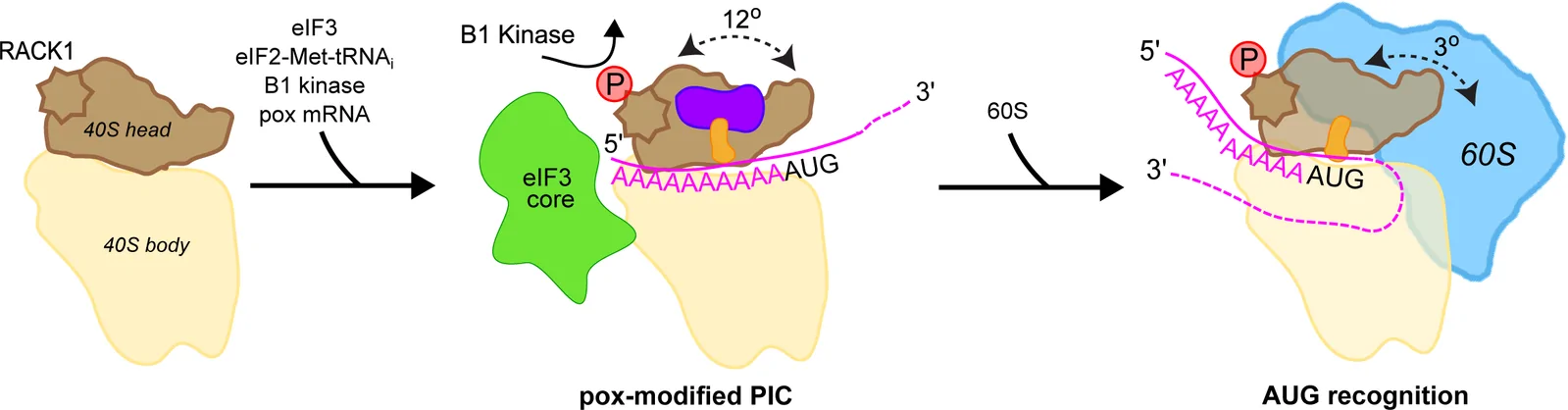
Poxvirus customization of ribosomes. Phosphorylation of the RACK1 linker by poxvirus B1 kinase creates electrostatic repulsion against the 18S rRNA, which broadens the range of motion of the 40S head akin to the effects of IRESs in Fig. 5. This promotes non-canonical initiation on short, unstructured viral mRNAs that have 5′ polyA leaders. RACK1 phosphorylation also increases the range of motion within the 40S head in 80S complexes to optimize AUG recognition on 5′ polyA-mRNAs.

Perhaps the best characterized form of ribosome customization by DNA viruses comes from the prototypical *orthopoxvirus*, vaccinia virus (VacV). The virus-encoded B1 kinase was implicated several decades ago in phosphorylating several RPs (124–126). More recently, these sites were identified using ribosome-specific proteomics in VacV-infected cells (118, 127) and global phosphoproteomics in monkeypox virus (MPXV)-infected cells (13). Among these, RACK1 has emerged as the primary target. RACK1 phosphorylation does not select against host transcripts but instead broadens the translational capacity of mammalian ribosomes to better accommodate non-canonical modes of initiation, including late viral mRNAs that contain unusual 5 polyadenylated (5′ polyA) leaders (127, 128). In mammals, ribosomes slip on homopolymeric adenosine sequences and, as such, polyadenylation is normally restricted to the 3′ UTR (28, 129, 130). Mechanistically, biochemical studies suggested that phosphorylation of RACK1 increases local electrostatic forces against 18S rRNA (131). In line with this, cryo-EM revealed that expression of a phosphomimetic of virally modified RACK1 increases the range of 40S head swivel and is sufficient to enhance translation of reporters with polyA sequences in either their 5′ UTR or coding region (127, 128, 131) (Fig. 6; Movie S2). In the context of infection, analysis of sub-classified states further showed that VacV specifically increases the range of 40S head conformations as part of the eIF3-containing PIC, but not in free 40S particles (132). Both eIF3 and RACK1 are linked to controlling 40S head movement, and functionally, both are required for efficient late viral protein synthesis (132–134), while an m^7^G-cap or the cap-binding protein eIF4E is not (135). Combined, these findings highlight how poxviruses use phosphorylation of RACK1 to control 40S head rotation to support simpler modes of initiation on their short, unstructured leaders. Finally, quantitative proteomics and cryo-EM show that VacV does not alter the RP composition of ribosomes, but RACK1 phosphorylation also increases 40S head rotation in assembled 80S ribosomes (128, 134). Accompanying RP dependency screens further revealed that beyond RACK1, late VacV mRNAs also rely on the normally dispensable P-stalk subunit, P2 (134). This suggests that 40S head closure and 60S joining are also fundamentally remodeled during infection to accommodate initiation on viral mRNAs (Fig. 6).

How RACK1 is phosphorylated has interesting evolutionary implications for both poxviruses and ribosomes. B1 phosphorylates a serine- and threonine-rich “S^276^T^277^S^278^S^279^” motif within a short linker that connects two of the seven β-sheets in RACK1. Only one residue is phosphorylated each time, with S^278^ being most abundantly detected for VacV (118, 127) and S^276^ for MPXV (13), although this difference may simply reflect technical challenges in mapping modifications in clustered motifs of this kind. Biochemical and functional studies show that single- or multi-site phosphomimetics enhance translation, but multi-site modifications create increasing electrostatic forces that reduce RACK1 affinity for the ribosome (127, 131). This suggests that poxviruses are exquisitely careful in only phosphorylating single sites on RACK1 to enhance translation while limiting deleterious effects on ribosome binding. While RACK1’s β-propeller domains mediate its interactions with other proteins, this linker is considered dispensable. Indeed, it is highly variable in length and amino acid sequence across species (127, 131). Mammalian RACK1 linkers do not contain negatively charged amino acids, but those of dicot plants and protists do, and these species often also limit clustered charge organizations. In agreement with earlier charge-affinity comparisons between human RACK1, poxvirus phosphomimetics, and human RACK1 chimeras with dicot/protist-like linkers (131), studies report electrostatic differences in how protist and human RACK1 interact with ribosomes (136). Moreover, dicots use RACK1 to control cap-independent translation of purine/A-rich leaders (137, 138), some protists encode widespread polyA leaders (139), both dicots and protists use A-rich AUG contexts (140), and protist ribosomes do not slip but, instead, tolerate adenosine tracts in coding regions (141). This is noteworthy because ancestral poxviruses are thought to have originated from protist NCLDVs (142). As such, poxviruses may have originated in organisms whose ribosomes accommodate polyA tracts, and after jumping into the animal kingdom, extant poxviruses evolved a kinase to convert mammalian ribosomes to a more protist-like state through carefully controlled RACK1 phosphorylation.

Finally, some RNA viruses can cause PTMs, but they remain less well characterized. For example, binding of the HCV IRES causes changes in RPS25 methylation (143), but whether this influences translation remains unknown. Similarly, whether RNA viruses actively regulate rRNA methylation to control translation is not widely reported, but rRNA 2′-O-methylation and pseudouridylation influence 40S head rotation, translational specificity, and efficient initiation by HCV and CrPV IRESs (144–146).

### Exploitation of pre-existing ribosome heterogeneity

Some RNA viruses exploit specific RPs and pre-existing ribosome heterogeneity in their host. Members of the Mononegavirales family, which have negative-strand ssRNA genomes and include rabies, measles, and vesicular stomatitis virus (VSV), utilize cap-dependent translation that requires RPL40 (147). RPL40 is dispensable for most host transcripts but enhances translation of stress-related mRNAs, and VSV co-opts host stress responses to recruit RPL40 to the ribosome. Moreover, while normally in stoichiometric equilibrium, cross-linking mass spectrometry suggests that under these stress conditions, a second copy of RPL40 is recruited to the beak region of the 40S head, near the mRNA entry channel to enhance translation of viral or stress-related mRNAs (148). While this intriguing observation has not yet been structurally confirmed, targeting of the 40S head again emerges as a theme and suggests that the repertoire for modifying ribosomes may extend to generating “super-stoichiometric” RPs during stress. Other RNA viruses exploit pre-existing heterogeneity. Cross-linking quantitative proteomics shows that RPS25 and RPL10A are present on ribosomes at sub-stoichiometric levels in mouse embryonic stem cells (40). Notably, the 60S subunit protein RPL10A interacts with and is required for efficient translation by the CrPV, HCV, and EMCV IRESs. Its position at the base of the L1 stalk near the E-site aligns with a role for RPL10A in IRES-mediated initiation, suggesting these viruses may be more reliant on specialized subsets of ribosomes than host mRNAs. Others, such as poliovirus, an enterovirus, and flaviviruses such as Zika and dengue virus, do not affect the ribosome itself but rewire its repertoire of associated proteins, including recruitment of proline hydroxylation machinery for co-translational folding of viral polyproteins (149).

## ELONGATION AND REINITIATION

During elongation, many RNA and DNA viruses use rare codons that require re-optimization for exogenous expression of their ORFs from plasmids. While in the context of infection, this can be intentional to couple translation rates with proper co-translational protein folding, recent studies suggest that many viruses evade the negative effects of codon bias by virtue of their 5′ UTRs and avoiding circularization of their mRNAs, which is driven by 5′ cap-3′ polyA tail interactions within host transcripts (150) (Fig. 4). This highlights how communication between the untranslated termini of transcripts also influences the decoding process and can be exploited by viruses.

**Fig 7:**
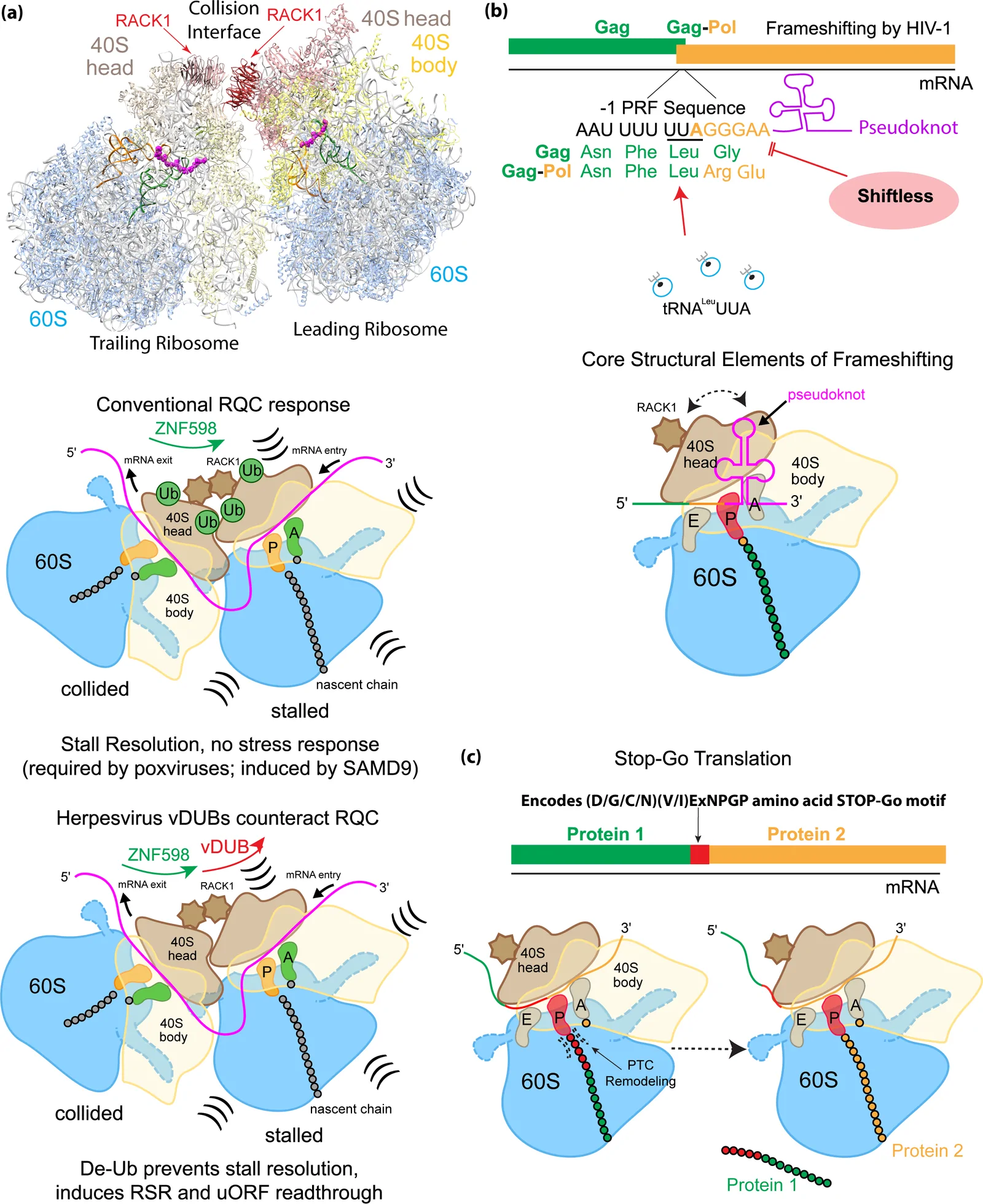
Regulation of ribosome collisions, frameshifting, and Stop-Go translation during elongation. (**a**) Ribosome stalling can result in collision with a trailing ribosome, forming a defined disome interface. A reference cryo-EM structure of two collided ribosomes is shown (top) to anchor the schematic representations beneath. These interfaces, centered around the 40S head and the scaffold protein RACK1, are recognized by factors such as ZNF598 and ZAK1α, acting as signaling hubs that initiate ribosome quality control (RQC) or the ribotoxic stress response (RSR). Poxviruses lack viral DUBs (vDUBs) and rely upon conventional RQC factors such as ZNF598 to resolve stalled, collided ribosomes. Stalling is induced as part of a poxvirus-specific antiviral response mediated by SAMD9, which depletes tRNA^Phe^. Herpesviruses, on the other hand, encode vDUBs that counteract ubiquitination by ZNF598, inducing the RSR wherein stalled ribosomes eventually read through stall sites. (**b**) Frameshifting allows HIV-1 to generate Gag and Gag-Pol fusion proteins through a −1 programmed ribosome frameshift (PRF) sequence where slippage occurs on a leucine-encoding UUA sequence, facilitated by a downstream pseudoknot that stalls ribosomes at the slippage site. Levels of tRNA^Leu^_UUA_ influence the efficiency of slippage and frame selection. The interferon-inducible protein shiftless inhibits frameshifting. Right: illustration of core frameshifting elements, including pseudoknots that interact with the A-site and entry channel, effects on P-site tRNA configuration and changes in both 40S head movement and positioning of RACK1, assisting with −1 slippage on adjacent slippery sequences. (**c**) Stop-Go translation by FMDV produces two proteins from one mRNA without a conventional stop-codon event or use of an RNA element. Instead, a (D/G/C/N)(V/I)ExNPGP amino acid motif in the nascent peptide (red) interacts with the exit tunnel resulting in hydrolysis of the nascent peptide without a stop codon. This releases the first encoded protein while continuing to translate the remaining ORF into a second protein (orange).

Beyond the core conformational dynamics of actively translating ribosomes, viruses must also contend with ribosome stalling and collisions, as well as regulated termination. Ribosomes can stall on structured RNAs, damaged templates, rare codons, or inhibitory nascent peptide sequences, forming the basis of the ribosome quality control (RQC) pathway that senses abnormal transcripts or translation events (28). This includes translation of homopolymeric adenosine stretches that cause mammalian ribosomes to slide and ultimately stall (129, 130). Sliding on these homopolymers senses aberrant internal polyadenylation or missing stop codons, which lead to readthrough of active ribosomes into the 3′ polyA tail. Trailing ribosomes then collide with stalled ribosomes and form structural states with distinct interfaces between adjacent 40S subunits (Fig. 7a). Central to this is the scaffolding protein, RACK1 on the solvent face of the 40S head, which stabilizes these states and facilitates their recognition by regulatory factors (151–153). Recognition by the E3 ligase, ZNF598 results in ubiquitination of RPS10 and RPS20, triggering RQC to resolve stalled ribosomes and degrade the problematic transcript and nascent peptide (151, 152, 154–156). When collisions are persistent or widespread, they activate ZAKα and the broader ribotoxic stress response (RSR) (153, 157, 158).

Termination and ribosome recycling similarly exploit the ribosome’s intrinsic motions. Stop-codon recognition by eRF1 and eRF3 is coupled to remodeling of both the decoding center and the PTC, triggering hydrolysis of the peptidyl-tRNA bond. Subsequent recycling involves large-scale rearrangements of the ribosome, including intersubunit rotation and head swiveling, which are harnessed by recycling factors to dissociate ribosomal subunits and reset them for additional rounds of translation (159–161). Although termination is often discussed separately from initiation and elongation, these stages form a continuous cycle, and their coupling is particularly relevant for viral strategies aimed at expanding coding capacity.

### Frameshifting and reinitiation

Viruses control elongating ribosomes through combinations of structured RNA elements that capture the ribosome itself and nearby slippery sequences that reposition it (49). Frameshifting involves slippage of ribosomes in −1 or +1 directions to create a new coding context and extended fusion protein. For example, HIV-1 uses a slippery −1 frameshift sequence and adjacent pseudoknot to control expression ratios of Gag and Gag-Pol fusion proteins from the same mRNA (162) (Fig. 7b). Cryo-EM shows the pseudoknot engages the A-site, hindering elongation and stimulating frameshifting on upstream U-rich sequences (163). HIV-1 frameshifting is sensitive to depletion of RPL4 (164) and is further influenced by leucine tRNA^Leu^_UUA_ levels that decode the UUA codon at the frameshift site. tRNA^Leu^_UUA_ is rare in human T-lymphocytes, the abundance of which regulates the rate of frameshifting and may influence HIV-1 replication efficiency in different cell types (165). Cryo-EM studies of other retroviruses show how these viral elements form dynamic “butterfly-like” double-pseudoknots (166), underscoring the structural and dynamic complexity of frameshifting as a whole. Interestingly, the interferon-inducible host protein, shiftless inhibits HIV-1 frameshifting (167). This antiviral activity likely evolved from shiftless’ evolutionarily earlier role as a low-abundance protein that clears rare frameshifted ribosomes from extremely long host mRNAs (168).

Coronaviruses use similar frameshift strategies, and their pseudoknot extensively remodels ribosomes as it captures and reprograms them (169–171). This includes interactions at the mRNA entry channel and altered 40S head movement, accompanied by a shift in RACK1 positioning (171). Additional interactions occur with tRNAs in the decoding center, including deformation of the P-site tRNA to facilitate the frameshift event. More broadly, −1 frameshift signals are diverse in structure and function (49) and include those that operate together with E-site tRNA modifications across several genera of RNA viruses, including *coronaviridae* and *retroviridae* (172). Moreover, studies in yeast suggest that defects in rRNA processing, common in several viral infections, can result in ribosomes with lower tRNA affinities and peptidyl-transferase activity, increasing stalling and enhancing −1 frameshifting (173).

Re-initiation involves sequences upstream of a stop codon that capture terminating ribosomes to restart at an adjacent ORF, creating a separate protein from a compact or overlapping ORF arrangement. While RNA structures and nucleotides involved in regulating these events have been extensively characterized (49), recent cryo-EM studies reveal how the rabbit hemorrhagic disease virus re-initiation element interacts with an 18S rRNA expansion sequence extending from the 40S head (174). Moreover, the reinitiation element occupies a range of positions, leading to conformational heterogeneity reflecting the dynamics of ribosome capture and control.

Finally, foot and mouth disease virus uses a “Stop-Go” event that produces two different proteins from an ORF without canonical termination or the use of an RNA element. Instead, a (D/G/C/N)(V/I)ExNPGP amino acid motif encoded in the nascent peptide itself interacts with the exit tunnel and reconfigures the PTC to induce hydrolysis and release of the first protein while continuing with translation of the ORF to produce a second protein (175) (Fig. 7c).

### Ribosome collisions and RQC

Interestingly, RNA viruses are not widely reported to induce stalling or ubiquitination events associated with RQC. This may reflect the fact that many RNA viruses encode vDUBs, which may counteract collision sensors like ZNF598. Moreover, RNA viruses carefully control frameshifting, which may limit ribosome collisions to begin with. Some RNA viruses also exploit the intrinsic functions of the P-stalk to prevent stalling on their mRNAs. The P1 and P2 proteins that form the stalk protrusion are largely dispensable for translation of many cellular mRNAs, yet they are required for efficient elongation on flavivirus ORFs by mitigating pausing at rare codons (176). Although the structural basis for this activity remains unclear, these findings highlight a selective role for the P-stalk in regulating translation and preventing pausing or stalling on specific mRNA subsets, including those of some flaviviruses and poxviruses (134, 176). These findings align with recent studies showing that the P-stalk recruits regulatory factors to selectively control translation during cellular stress and cytokine responses (for example, see references 177–179). Finally, hepatitis A virus (HAV) induces and requires a ubiquitin-like modification, termed UFMylation of the 60S protein, RPL26 (180). RPL26 UFMylation is associated with a specialized form of RQC that occurs at the endoplasmic reticulum (ER) (181–183), which is extensively remodeled into picornavirus replication compartments. However, other picornaviruses tested do not show the same dependency, suggesting that RPL26 UFMylation may instead be important for translation from the HAV IRES (180). Overall, there are limited reports to date connecting collisions and RQC with RNA virus infections.

By contrast, several DNA viruses rely upon or actively regulate RQC and RSR pathways. This includes herpesviruses, which encode vDUBs that counteract ubiquitination of RPS3, RPS10, and RPS20 (184–186). This prevents the resolution of stalled ribosomes, leading to a stress response and eventual read-through events that promote translation of uORFs and from IRES elements (186). This is notable considering that herpesviruses are among the few DNA viruses reported to use IRES elements, albeit in only one or two of their many transcripts. Most of these vDUBs also counteract UFMylation of RPL26 (185), which is likely an important dual function given the extent of ER stress induced by viral replication. However, there is divergence in this dual functionality as the HSV-1 vDUB counteracts RQC-related ubiquitination but cannot counteract UFMylation (184). Beyond RQC, the ubiquitin–RP fusion proteins, Ub-RPS27a and Ub-RPL40, are degraded if they are not processed by host DUBs (44, 45). However, recent studies in human cells revealed that distinct DUB types can alter this fate and can lead to stabilized extra-ribosomal fusion proteins (45). It would be interesting to determine whether beyond RQC, herpesvirus vDUBs also influence this or the broader homeostatic pathways that normally degrade extra-ribosomal RPs.

Stalled ribosomes play a different role in poxvirus infection, with interesting evolutionary and antiviral implications. Unlike herpesviruses, poxviruses only encode inactive remnants of vDUBs and rely upon ZNF598 for efficient translation (187, 188). Initial studies of a limited number of viral proteins led to suggestions that ZNF598 might independently regulate initiation on late viral transcripts with 5′ polyA-leaders (187). However, subsequent proteomic studies found that RPS10 and RPS20 ubiquitination is stimulated very early in infection, and that ZNF598 enhances translation of both early and late poxvirus mRNAs, which have very different 5′ UTRs (188). Recently, the poxvirus restriction factor, SAMD9, was found to act as an anticodon nuclease (ACnase) that cleaves phenylalanine tRNA (tRNA^Phe^) (189). While this activity is silent in uninfected cells, it is activated upon poxvirus infection and causes codon-specific ribosome stalling and stress signaling. Although poxviruses encode antagonists of SAMD9, emphasizing this pathway’s importance, they likely further benefit from ZNF598-mediated resolution of collisions to maximize the pool of active ribosomes and reduce the overall severity of this antiviral response. Cells may have evolved this virus-specific approach to activating RQC due to the way in which poxviruses structurally remodel ribosomal interactions with polyA sequences, which includes increased readthrough of polyA stall reporters (128, 188). Indeed, some protists do not activate RQC on polyA sequences (136, 141), suggesting that this may represent a species- and RNA sequence-specific antiviral response pathway that poxviruses cleverly rewire in humans.

On this point, many viruses disrupt host mRNA processing and cause aberrant polyadenylation events, but whether this activates polyA-based RQC pathways as part of an antiviral response remains understudied. Notably, ZNF598 independently represses interferon-stimulated gene (ISG) expression (187, 190), and its sequestration to infection-induced ribosome collisions may, in turn, enhance ISG responses. Such pathways may also be species-, RNA sequence-, and virus-specific. For example, ZNF598 enhances infection by VacV but not the RNA virus, VSV, in human cells (187), while in plants, pelota, a conserved ribosome rescue factor in RQC, similarly benefits DNA viruses but restricts RNA viruses (191). Moreover, cyclic GMP-AMP synthase (cGAS), a potent DNA sensor and antiviral effector, is maximally activated upon binding collided ribosomes (192), but how this operates during infection remains to be determined. Combined, these findings provide tantalizing evidence that ribosome collisions function as underappreciated hubs in sensing infection and amplifying antiviral responses.

## CONCLUDING REMARKS

Technological advances in cryo-EM, genomics, and proteomics have led to a remarkable shift in our appreciation of ribosome heterogeneity and conformational dynamics as direct targets for viral control of translation in their mammalian hosts. In particular, the solvent face of the 40S subunit is emerging as a key regulatory platform for remodeling the mRNA channel to control virtually every step in the translation process, and recent studies highlight how this extends to broader biological processes (193). With many viruses still uncharacterized, our understanding will undoubtedly evolve in yet more exciting directions, and one can only wonder whether the 40S head remains a central theme, or whether unusual strategies such as tRNA mimicry emerge as more widespread. From a mechanistic perspective, it is important to note that some IRESs, tRNA mimicry strategies, and frameshifting elements have been shown to directly control ribosome dynamics to, in turn, control translation. Moreover, certain PTMs such as RACK1 phosphorylation by poxviruses have also been shown to alter ribosome structure and function irrespective of infection or the viral or host RNA species being translated. However, the simple observation of altered structures is not proof of control over the ribosome and should be interpreted cautiously in the absence of complementary functional evidence, as such differences can also simply reflect the alternative mode of translation itself rather than active structural remodeling. Understanding the structural basis of these highly specific modes of translation also offers opportunities to develop new antivirals targeting, for example, IRES-mediated initiation or frameshifting by pandemic viruses including HIV-1 and SARS-CoV-2 (194–197).

While we offer cautionary notes on extra-ribosomal functions of RPs in cell lines, several viruses encode vDUBs. This includes herpesviruses that are associated with cancers, suggesting some viruses may induce extra-ribosomal populations of RPs as part of their natural replication strategy and cellular transformation capacity, a challenging but important question in the field. Moreover, considering that vDUBs influence a wide range of processes, the specific contribution of their effects on RQC and RP homeostasis to viral replication remains to be fully elucidated. On a related note, while viruses modify ribosomes to their own ends, this often occurs in the absence of global changes in RP composition. However, it would be interesting to determine whether cell type-specific expression of RPs is also a determinant of cellular tropism, akin to how tissue-specific RPL38 expression influences HOX mRNA translation and cytoskeletal patterning during development. Clearly, our understanding of how ribosome heterogeneity is controlled and influences infection outcomes remains in an exciting, early stage.

## AFTER EPUB

*[This article was published on 14 September 2026 with a preprint cited as reference 148. The reference was updated in the current version, posted on 8 October 2026.]*

## SUPPLEMENTAL MATERIAL

Movie S1: Ribosome dynamics in uninfected cells jvi.01282-26-s0001.mp4 Structure-based animation illustrating key conformational motions of the human ribosome during (i) initiation, which involves 40S head swiveling and opening to accommodate mRNA loading and scanning, and (ii) elongation, which involves 40S head swiveling and inter-subunit rotation, together with coordinated tRNA movement. eIFs are omitted so that ribosome movements are clearly visible. Structures used to inform this animation, and those in Movie S2, include PDB 6Z6M, 4UG0, 5FLX, 6ZVJ, and 6ZOJ and EMDB entries 24542, 24548, and 46447.

Movie S2: Virus-induced remodeling of ribosome dynamics jvi.01282-26-s0002.mp4 This structure-based animation highlights some key examples of the diverse strategies used by viruses to reshape ribosome dynamics, which include RNA-mediated remodeling of the 40S head by IRESs, phosphorylation of RACK1 to alter 40S head swivel by poxviruses, and coronavirus Nsp1-mediated occlusion of the mRNA entry channel that selectively allows viral mRNAs to access 40S subunits for initiation.

ASM does not own the copyrights to Supplemental Material that may be linked to, or accessed through, an article. The authors have granted ASM a non-exclusive, world-wide license to publish the Supplemental Material files. Please contact the corresponding author directly for reuse.
